# Neuroimmunomodulation induced by mud-bath therapy: clinical benefits and bioregulation of the innate/inflammatory responses induced by a peloid enriched with rosmarinic acid in elderly patients with osteoarthritis

**DOI:** 10.1007/s00484-025-02984-7

**Published:** 2025-07-16

**Authors:** Eduardo Ortega-Collazos, Eduardo Otero, Casimiro López-Jurado, Carmen Navarro, Leticia Martín-Cordero, Isabel Gálvez, Silvia Torres-Piles, Eduardo Ortega, María Dolores Hinchado

**Affiliations:** 1 Immunophysiology Research Group, Instituto Universitario de Investigación Biosanitaria de Extremadura (INUBE), University of Extremadura, Badajoz, Spain; 2 Department of Internal Medicine, Hospital Universitario de Cáceres, Cáceres, Spain; 3 Departament of Nursing, Facultad de Medicina y Ciencias de la Salud, University of Extremadura, Badajoz, Spain

**Keywords:** Balneotherapy, Inflammation, Stress, Phagocytosis, Peloids, Rosmarinic acid

## Abstract

Balneotherapy using peloids (mud-bath therapy) is one of the most effective non-pharmacological approaches for managing osteoarticular and musculoskeletal disorders, such as osteoarthritis (OA). Recent studies have shown that peloids enriched with rosmarinic acid (RosA) enhance the therapeutic effects of mud therapy in elderly OA patients. The objective of this study was to evaluate whether the normalization of the interaction between the hormetic stress response, mediated by cortisol, and the inflammatory response, mediated by IL-8 (measured in blood by ELISA), underlies the clinical benefits of a hyperthermic mud-bath intervention using a RosA-enriched peloid, a polyphenolic acid with anti-inflammatory and immunomodulatory properties. Additionally, we aimed to investigate whether this neuroimmune regulation contributes to enhancing the innate immune response, specifically the phagocytic and microbicidal activity of neutrophils (assessed by flow cytometry). Twenty-three elderly OA patients participated in the mud-bath intervention, a 10-day cycle of hyperthermic balneotherapy using a RosA-fortified peloid and mineral medicinal water at 40 °C. Significant increases in systemic cortisol levels, along with a notable decrease in IL-8 and enhanced phagocytic and microbicidal activity of neutrophils, were observed following the intervention. These effects were accompanied by improved knee mobility and reduced pain. In conclusion, hyperthermic mud-bath therapy with a RosA-enriched peloid induces immunoneuroendocrine stabilization, which underlies the clinical benefits of the balneological intervention. Finally, concepts such as neuroimmunomodulation, hormesis, and the bioregulation of the innate and inflammatory immune responses in the context of balneotherapy are also discussed.

## Introduction

Traditional pharmacological treatments for osteoarthritis (OA), such as nonsteroidal anti-inflammatory drugs (NSAIDs) and corticosteroids, primarily focus on symptom management rather than modifying the disease’s progression. In contrast, within the realm of complementary and conservative therapies, balneotherapy—often combined with mud therapy—has emerged as a widely utilized non-pharmacological treatment for OA. Numerous studies conducted over the years have provided strong evidence supporting the efficacy and safety of balneotherapy and mud therapy in enhancing functional capacity and reducing pain in OA patients (Harzy et al. 2009; Espejo Antúnez et al. 2013; Fioravanti et al. 2015; Aydin et al. 2024). However, despite these clinical benefits, the mechanisms underlying their therapeutic effects remain inadequately understood, hindering their broader acceptance and integration into mainstream medical practice. Therefore, it is crucial to acknowledge that the pathogenesis of OA is far more complex than the traditionally viewed concept of a simple degenerative, wear-and-tear process (Galvez et al. 2017; Van den Bosch et al. 2020; Katz et al. 2021).

Although the precise multifactorial pathophysiological mechanisms underlying OA remain largely unknown, it is well established that various inflammatory and immune processes play a significant role in its pathogenesis, progression, and overall impact (Rahmati et al. 2016; Galvez et al. 2017; Van den Bosch et al. 2020; Tognolo et al. 2022). In addition to localized inflammatory events within joint tissues, such as the release of inflammatory mediators by cartilage, bone, and synovium (Kapoor et al. 2011; Van den Bosch et al. 2020), low-grade systemic inflammation has emerged as a critical factor in the pathogenesis of OA (Galvez et al. 2017). Thus, this systemic inflammation may not only initiate but also exacerbate the progression of OA. Moreover, locally produced inflammatory mediators, including cytokines, can be detected in peripheral blood, thereby sustaining and amplifying the systemic inflammatory response (Berenbaum 2013; Galvez et al. 2017; Cheleschi et al. 2022). Additionally, immune-neuroendocrine dysregulation has been observed, disrupting the negative feedback between the inflammatory and stress responses, along with a reduction in the functional capacity of phagocytes. This reflects a disturbed regulation of innate immune/inflammatory responses and a suppression of the immune system’s defenses against pathogens (Galvez et al. 2017).

Research conducted by our group has demonstrated that mud therapy may exert anti-inflammatory effects in OA patients through the modulation of systemic cytokines, thereby enhancing immune-neuroendocrine regulation. This modulation may contribute to the alleviation of symptoms in OA (Ortega et al. 2017). However, it is important to recognize that the inflammatory response is an integral component of the innate immune system. Consequently, the anti-inflammatory effects induced by mud-bath therapy could potentially compromise the innate immune response to pathogens. In fact, a study has shown that the anti-inflammatory mechanisms underlying the benefits of hyperthermia induced by mud-bath therapy may also reduce the oxygen-dependent microbicidal activity of phagocytic cells, such as monocytes (Gálvez et al. 2020). Therefore, it is crucial to develop balneological strategies that reduce sterile inflammation in rheumatic diseases without compromising the innate immune response against pathogens, thereby preventing increased susceptibility to infections in patients.

A well-balanced neuroimmune regulation between the inflammatory response mediated by IL-8, a cytokine directly associated with pain in OA patients (Gálvez et al. 2018b), and the stress response mediated by cortisol, may underlie the clinical benefits of mud-bath therapy in elderly OA patients (Ortega et al. 2017; Gálvez et al. 2018b). Furthermore, a recent pilot study has suggested that mud-bath therapy using a peloid enriched with Rosmarinic Acid (RosA) provides similar clinical benefits to that of a non-supplemented peloid. However, in contrast to the non-enriched peloid, the RosA-enriched treatment also appears to enhance the innate immune response against pathogens (Ortega-Collazos et al. 2024). Due to its anti-inflammatory properties, RosA has been utilized as a natural therapeutic agent in OA patients administered orally and explored within the context of food science (Connelly et al. 2014; Guan et al. 2022). However, the potential anti-inflammatory effects of RosA in the context of balneotherapy have not been fully explored. It is plausible to think that the incorporation of RosA into mud treatments could prevent a potential immunosuppression of the innate immune response against pathogens in the context of the anti-inflammatory effects of mud-bath interventions in elderly OA patients.

The objective of the present investigation was to assess whether the normalization of the regulatory interactions between the systemic inflammatory response mediated by IL-8 and the hormetic stress response mediated by cortisol can immunophysiologically mediate the clinical benefits of a mud-therapy intervention using a peloid enriched with RosA. Additionally, it aimed to determine whether this potential immune-neuroendocrine regulation does not compromise the innate immune response against pathogens.

## Materials and methods

This intervention, involving pre- and post-mud baths, took place at the ‘El Raposo’ healthcare and spa center (Puebla de Sancho Pérez, Badajoz, Spain), which was designated a Public Utility in 1926. The impact of a mud therapy cycle was assessed using mud that had been artificially enriched with RosA, in a group of patients with osteoarthritis (OA) participating in the Social Thermalism Program organized by the Elderly and Social Services Institute (IMSERSO) of Spain.

A total of thirty-seven individuals with primary knee osteoarthritis (OA) were initially assessed for their suitability for the study. Each volunteer underwent a preliminary medical assessment to check for any potential contraindications to the experimental procedure. After being fully informed about the study, participants who met the set inclusion and exclusion criteria were enrolled. The inclusion requirements specified that participants should be at least 60 years of age and diagnosed with primary knee OA by a rheumatologist, following the American College of Rheumatology (ACR) guidelines (Altman et al. 1986). Additionally, participants were required to have no other conditions that would interfere with the experimental treatment. Based on the knee flexion angle recorded before the intervention, none of the participants showed extremely severe OA symptoms.

The exclusion criteria included the presence of any infections, cancerous conditions, or issues related to the cardiopulmonary, vascular, inflammatory, immune, or other musculoskeletal systems. Participants who had undergone total or partial knee replacement, received physical therapy in the six months prior to the study, or had received intra-articular injections of corticosteroids or hyaluronic acid within the last six months were also excluded. Additionally, individuals who had taken oral or local corticosteroids, anti-cytokine treatments, or any form of biological therapy were not eligible.

**Fig. 1:**
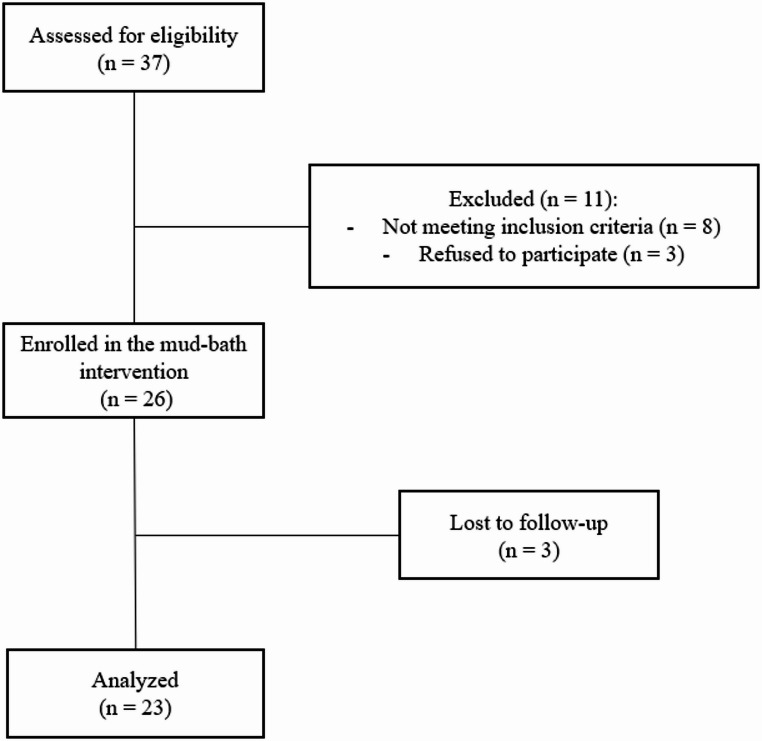
Flowchart of patients through the study

A total of twenty-six patients were initially included in the study after meeting the eligibility criteria and/or consenting to participate. Three patients were lost to follow-up, leaving twenty-three patients who were ultimately included for evaluation (Fig. 1).

Table 1 shows the anthropometric and clinical data. To ensure anonymity, each participant was assigned a unique alphanumeric code. Anthropometric measurements and clinical variables were assessed, and blood samples were collected between 8:00 and 9:00 a.m. The baseline evaluation and sampling took place the day after participants arrived at the spa center, before the first session of mud therapy. Post-treatment samples were collected one day after the final mud therapy session, to prevent any effects from acute interventions being included in the evaluation. The study received approval from the Ethical Committee of the University of Extremadura, Spain, following the guidelines established by the European Community Council Directives and the Helsinki Declaration (Reg. No. 96/2022 and Reg. No. 146/2024).of the final volunteers participating in the study.

**Table 1:** Anthropometric and clinical data in OA patients participating in the study before and after the mud-bath intervention

*n* = 23	Pre-intervention	Post-intervention
Sex (%)	Women (60.8%)	
Ethnic group (%)	Caucasian (100%)	
Age (years)	71.6 ± 1.4	
Weight (kg)	75.1 ± 2.7	75.3 ± 2.6
BMI	29.4 ± 0.8	29.1 ± 0.8
Fat mass (%)	37.7 ± 1.1	37.4 ± 1.1
Muscle mass (kg)	44.1 ± 1.6	45.0 ± 1.6
Bone mass (kg)	2.3 ± 0.1	2.3 ± 0.1
Body water (%)	43.4 ± 0.7	43.0 ± 1.6
Basal metabolism (kcal)	1400.2 ± 47.1	1411.6 ± 51.7
*Mobility*		
Average knee flexion angle (º)	112.1 ± 2.0	120.1 ± 2.3 **
Average knee extension angle (º)	171.1 ± 1.6	175.2 ± 1.2 *
*Pain*		
VAS	4.2 ± 0.6	1.8 ± 0.4 ***
OA patients taking analgesic (%)	60.10%	30.40%

*BMI* Body Mass Index, *VAS* Visual Analogic Scale. * *p* < 0.05, ** *p* < 0.01, ***p < 0.001 compared with the corresponding pre- intervention values. Some anthropometric (BMI) and the pain data from these patients correspond to a study group within a broader research project, previously used in earlier studies (Ortega-Collazos et al. 2024), whose biological samples were preserved for the immunoneuroendocrine analyses conducted in the present investigation

### Intervention

Artificially-matured mud is a solid/liquid content mixture at controlled proportions, in order to obtain a final product with the same basic properties of natural mud. Briefly, it consists of a blend of bicarbonate-rich mineral-medicinal water from the spring at the ‘El Raposo’ spa center, a clayey-silt solid phase, with a microalga, and with RosA. The water of “El Raposo” spa is hypothermal, very hard, of medium mineralization; and contains bicarbonate (396.5 mg/L) and calcium (130.2 mg/L) as predominant ions. The mineral-medicinal water fraction represents a 32% approximately of the mud. The major chemical elements of the muds are SiO_2_, CaO, Al_2_O_3_ and Fe_2_O_3_. (Carretero et al. 2010; Pozo et al. 2013; Ortega et al. 2017).

#### Peloid preparation

Peloids were provided by the research group of Prof. Legido from the University of Vigo (Spain). The starting point was the sifted solid substrate, which was subjected to a drying process in an oven at 110 °C. The biological content, derived from the microalga *Monoraphidium pusillum*, is sourced from the natural spring and cultivated separately in photobioreactors. The microalgae were incorporated into the mixture in the aqueous phase, as obtained from the photobioreactor, with a controlled concentration of 1%. As a result, the mud undergoes a controlled maturation process, lasting at least two months. Once the solid and liquid phases were mixed (approximate ratio 35:65), the maturation process was carried out. After maturation, 0.5% rosmarinic acid (Sigma-Aldrich, CAS number 20283-92-5), a bioactive compound, was added to the mixture.

Patients underwent daily mud therapy sessions for 10 consecutive days, adhering to the therapeutic protocol recommended by their healthcare providers. Each session involved the application of mud at 40–42 °C using a brush to the entire body, followed by a drying period of 45–60 min in a solarium. Afterward, participants took a mud bath, which consisted of a mixture of mineral-medicinal water and mud, at a temperature of 38–40 °C for 15 min. The session concluded with the removal of mud remnants using a thermal water jet at 38–40 °C for 2 min. No adverse events were reported.

### Anthropometric and clinical data

Body weight, body fat percentage, muscle mass (kg), bone mass (kg), body water percentage, and basal metabolic rate (kcal) were assessed using a bioimpedance scale (BIA TANITA DC-360, Tokyo, Japan). The body mass index (BMI) was determined using the formula: BMI = weight (kg)/height² (m²). Knee flexion and extension angles were assessed with a goniometer. Pain intensity was measured using a visual analogue scale (VAS).

### Whole blood extraction, determination of systemic concentration of inflammatory and stress biomarkers, and evaluation of the innate immune response

Peripheral blood samples were collected in a fasted state through sterile puncture of the antecubital fossa veins and placed in collection tubes for serum isolation. The samples were kept at room temperature for 15–20 min and then centrifuged at 1800 × g for 15 min. Serum samples were initially refrigerated at −20 °C and later stored at −80 °C until further analysis. Inflammatory cytokine concentrations (IL-8 and IL-6) and cortisol levels were measured using commercial ELISA kits (Diaclone SAS, Biotech. Inv. Group, Besancon Cedex; DetectX^®^, ArborAssays, Ann Arbor, MI, USA).

The phagocytic and oxygen-dependent microbicidal capacity (respiratory burst activity through O2- production) of neutrophils against opsonized *Staphylococcus epidermidis* was evaluated using flow cytometry on heparinized whole blood. This quantitative technique allows for the assessment of both the number of bacteria ingested and the production of O2 per cell by measuring the mean fluorescence intensity (MFI) of active phagocytic cells, which reflects the phagocytic and oxygen-dependent microbicidal activity of neutrophils (Galvez et al. 2017). Briefly, bacteria were labeled with fluorescein isothiocyanate (FITC) and opsonized with human serum. Blood samples were incubated with the opsonized bacteria, Hoechst 33,342, actinomycin D, PBS, and FBS. After 30 min, hydroethidine was added to detect O2- production, followed by an additional 30 min of incubation. The samples were subsequently analyzed using a flow cytometer (MACSQuant^®^ VYB, Miltenyi Biotec GmbH).

### Statistical analysis

The data are presented as the mean ± standard error of the mean (SEM). The normality of the variables was assessed using the Kolmogorov-Smirnov test, followed by Student’s t-test for paired samples. A significance level of *p* < 0.05 was considered statistically significant. Calculations were performed using the IBM^®^ SPSS^®^ Statistics version 22 software package.

##  Results

Anthropometric and clinical data before and after the mud-bath intervention in elderly OA patients are presented in Table 1. Regarding anthropometric data, no significant differences were found between pre- and post-intervention values in any of the measured variables, including BMI, fat mass, muscle mass, bone mass, body water, and basal metabolism. However, as expected, significant improvements were observed in the clinical variables, with a marked increase in both average knee flexion (*p* < 0.01) and average knee extension (*p* < 0.05) compared with baseline values prior to the intervention. This demonstrated a clear improvement in mobility following the mud-bath intervention with a peloid enriched with RosA and hyperthermia with mineral water. Furthermore, a significant reduction in pain (*p* < 0.001 relative to baseline values) as measured by the Visual Analog Scale (VAS) was observed, along with a 50% reduction in the number of patients requiring analgesics.

**Fig. 2:**
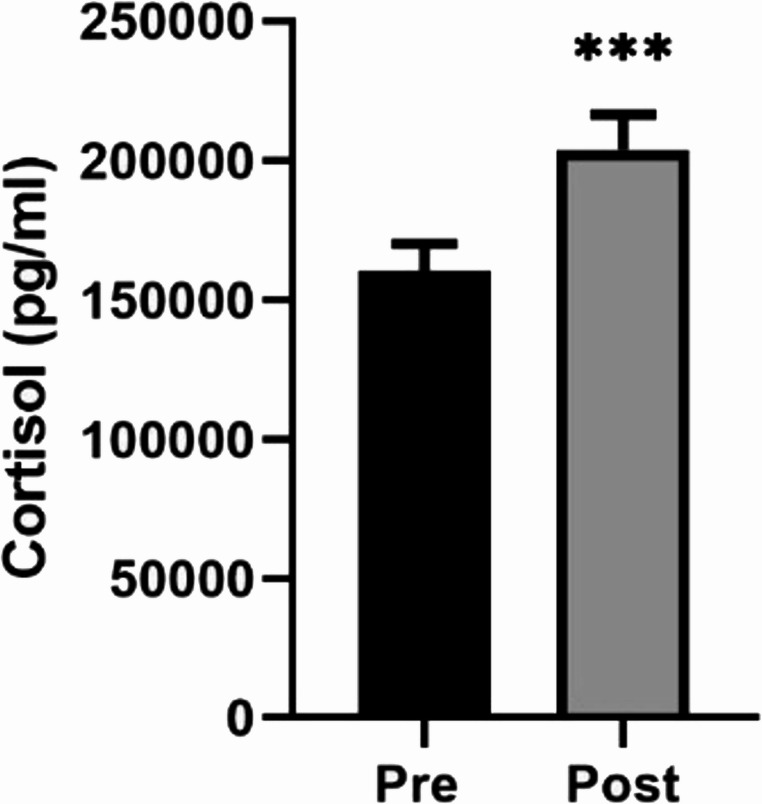
Effect of mud-bath intervention on the systemic concentration of cortisol (pg/ml). Each column represents the mean ± SEM of cortisol determination in the pre- and post-intervention situations *** *p* < 0.001 compared with the corresponding pre-intervention values

**Fig. 3:**
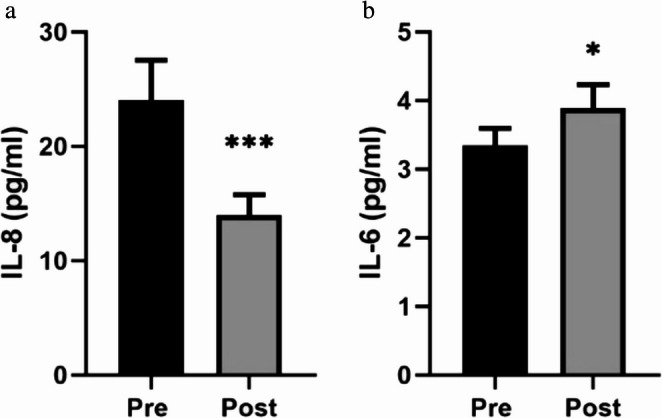
Effect of mud-bath intervention on the systemic concentration of IL-8 (**a**) and IL-6 (**b**). Each column represents the mean ± SEM of cytokine determinations in the pre- and post-intervention situations. * *p* < 0.05, *** *p* < 0.001 compared with the corresponding pre-intervention values

Figures 2 and 3 present the results corresponding to the effect of the mud-bath intervention on the stress response, as mediated by systemic cortisol concentrations, and the systemic inflammatory response, evaluated through the concentrations of IL-8 and IL-6. The effect on the physiological stress response was evidenced by a significant increase (*p* < 0.001 compared with pre- intervention values) in circulating cortisol levels (Fig. 2). The effect on the anti-inflammatory response was indicated by a decrease (*p* < 0.001 compared with pre- intervention values) in the concentration of the pro-inflammatory cytokine IL-8 (Fig. 3a), alongside an increase (*p* < 0.01 compared with pre- intervention values) in the concentration of IL-6, a regulatory cytokine involved in the inflammatory response (Fig. 3b).

**Fig. 4:**
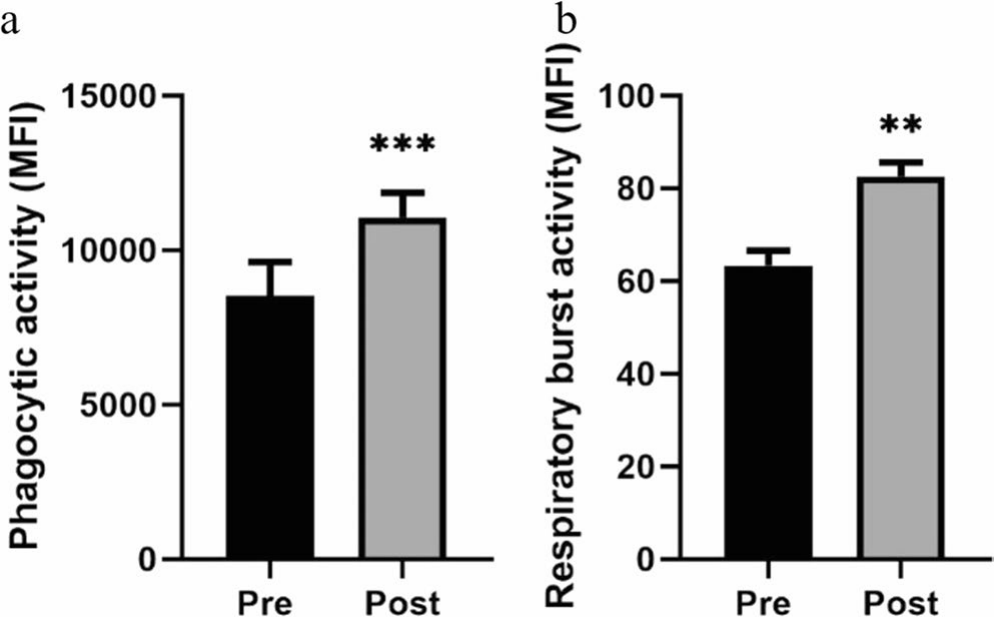
Effect of mud-bath intervention on the innate immune response: phagocytic activity (measured by the mean of fluorescence intensity (MFI)) (**a**) and oxygen-dependent microbicide activity (measured by the O₂⁻ production activity, MFI) (**b**). Each column represents the mean ± SEM of the values in the pre- and the post-intervention ** *p* < 0.01, *** *p* < 0.001 compared with the corresponding pre-intervention values

Finally, the decrease in the pro-inflammatory response does not lead to a parallel decline in the innate immune response mediated by neutrophils. On the contrary, the results presented in Figure 4 show an increase, compared with pre- intervention values, in both phagocytic activity (Fig. 4a; *p* < 0.001) and oxygen-dependent microbicidal activity (O₂⁻ production) (Fig. 4b; *p* < 0.01) following the mud-bath intervention.

##  Discussion

To the best of our knowledge, this is the first study evaluating the neuroimmune mechanisms underlying the clinical benefits of a peloid enriched with RosA following a mud-bath intervention in elderly patients with osteoarthritis (OA). In addition to the anti-inflammatory and clinical benefits of RosA reported in OA patients in the context of food sciences (Guan et al. 2022), recent studies have also demonstrated that peloids enriched with RosA enhance the clinical benefits of mud-bath therapy (Ortega-Collazos et al. 2024). Moreover, the present investigation shows that the hyperthermic mud-bath intervention with a RosA-enriched peloid induces a hormetic stress response, characterized by a physiological increase in cortisol concentration. This response may mediate the observed anti-inflammatory effects, as evidenced by a reduction in the systemic concentration of the pro-inflammatory cytokine IL-8, along with an increase in the concentration of the regulatory inflammatory cytokine IL-6. These changes underlie the improved mobility and reduced pain in OA patients, serving as a mechanism of therapeutic effectiveness. Furthermore, the anti-inflammatory effects induced by the hormetic stress following the mud-bath therapy with hyperthermic mineral water do not lead to an immunocompromised innate immune response. On the contrary, the RosA-enriched peloid stimulates the innate immune response, enhancing the phagocytic and microbicidal activity of neutrophils, thereby helping to prevent pathogen challenges. This is likely a differential effect induced by RosA enrichment in the peloid, as similar previous interventions without RosA did not show clear stimulatory effects on phagocytic and, particularly, microbicidal capacities (Gálvez et al. 2020; Ortega-Collazos el al. 2024).

Although the clinical benefits of balneotherapy in general (Tognolo et al. 2022) and mud-bath therapy or pelotherapy in particular (Espejo Antúnez et al. 2013; Fioravanti et al. 2015; Aydin et al. 2024; Ortega-Collazos et al. 2024) for patients with osteoarthritis (OA) have been clearly demonstrated, it was only in the past year that neuroimmune stabilization—mediated by the interaction between the stress and inflammatory responses, which are inherently dysregulated in elderly individuals with OA (Galvez et al. 2017)—was shown to underlie the immunophysiological mechanisms responsible for these clinical benefits (Ortega et al. 2017; Gálvez et al. 2018b, 2020). Most of the clinical benefits of balneotherapy interventions have been attributed to hyperthermia, which is also considered a form of hormetic stress. Hormesis refers to a biphasic dose-response relationship in which exposure to a low dose of a chemical or environmental factor induces beneficial stimulatory or adaptive effects, whereas higher doses result in inhibitory or toxic outcomes. This response to low doses of stress triggers an adaptive compensatory process or stress adaptation, following an initial disruption of homeostasis (Calabrese et al. 2007; Mattson 2008). Heat is classified as a physical hormetin, meaning it is a condition that can induce physiological hormetic effects by activating various pathways involved in protective adaptive stress responses (Rattan and Demirovic 2010). In this context, several studies provide compelling evidence that the beneficial effects of balneotherapy and hydrotherapy align with the concept of hormesis, thus supporting the role of hormesis in hydrothermal treatments. These interventions involve not only physical hormetins, such as heat, but also chemical components present in mineral waters, as well as psychological hormetins (Gálvez et al. 2018a).

One of the effects induced by physiological hormetic stress responses is an improved bioregulatory and adaptive interaction between the inflammatory and stress responses, particularly when these are dysregulated, as seen in pathologies involving low-grade inflammation, such as osteoarthritis (OA) (Galvez et al. 2017; Ortega et al. 2018). The bioregulatory responses of the innate immune/inflammatory system primarily involve the suppression of elevated basal inflammatory states or sterile inflammation, while simultaneously stimulating, or at the very least, not impairing the innate immune response to pathogens. These responses have been shown to be induced by optimal conditions of hormetic physiological stress, such as exercise (Ortega 2016) or mud-therapy interventions in OA patients (Ortega et al. 2017; Gálvez et al. 2018a, b). It is well established that the hypothalamic-pituitary-adrenal (HPA) axis is activated in response to both endogenous stressors (e.g., immune-mediated stress) (del Rey and Besedovsky 2000; Ortega 2016) and exogenous stressors (e.g., hyperthermia), ultimately leading to the release of cortisol (Møller et al. 1989; Ježova et al. 1994; Matsumoto et al. 2001; Gálvez et al. 2018a). The released cortisol is particularly important due to its anti-inflammatory effects and its ability to inhibit the production of additional inflammatory mediators, such as pro-inflammatory cytokines (del Rey and Besedovsky 2000; Gálvez et al. 2018a), thereby preventing sterile inflammation. Therefore, the reduction of sterile inflammation mediated by the elevation and adaptive normalization of cortisol levels has been proposed as a key mechanism for decreasing sterile inflammation in OA following mud-bath interventions (Ortega et al. 2017; Gálvez et al. 2018b).

Despite the demonstrated positive effects of balneotherapy on mental health, such as reducing psychological stress and anxiety, there is controversy regarding the role of cortisol levels in this context (Antonelli and Donelli 2018). Recently, Antonelli and colleagues (Antonelli et al. 2024) conducted an excellent review and meta-analysis of original studies, providing evidence that the regulation of the hypothalamic-pituitary-adrenal (HPA) axis contributes to the beneficial effects of mud-balneotherapy on health parameters and quality of life, preventing stress-related conditions in healthy individuals and alleviating symptoms in patients with chronic diseases. The data collected indicate a significant short-term reduction in cortisol levels in healthy adults undergoing balneotherapy, particularly in those with high stress levels. Conversely, in elderly patients with rheumatic conditions, mud-bath balneotherapy has been shown to increase cortisol levels (Ortega et al. 2017; Stanciu et al. 2017), which is consistent with the results obtained in the present investigation. Additionally, water balneotherapy (42 °C) with calcium and bicarbonate in elderly healthy individuals has also been shown to increase salivary cortisol (Toda et al. 2006), reinforcing the importance of aging, during which dysregulated neuroimmunomodulation has also been demonstrated (De la Fuente et al. 2011; Félix et al. 2024).

In addition to neuroimmunoendocrine stabilization, the beneficial effects of balneotherapy, with or without peloids, also extend to the modulation of the immune system (Gálvez et al. 2018b; Maccarone et al. 2021). Once it was determined that the physiological cortisol release induced by the hormetic stress of the mud-bath intervention improved the bidirectional interaction between the inflammatory and stress responses mediated by IL-8 and cortisol, we aimed to assess the effect of this intervention on the innate immune response to pathogens. This is particularly relevant, as the inflammatory response is a component of the innate immune system, and OA patients exhibit reduced phagocytic and microbicidal capacity against pathogens (Galvez et al. 2017). The results clearly demonstrated that the mud-bath intervention also significantly stimulates the phagocytic and microbicidal activity of neutrophils, confirming a clear bioregulatory effect on both the innate and inflammatory responses induced by this intervention. These findings are consistent with the evidence that physiological concentrations of glucocorticoids released during stress situations stimulate phagocytic activity (Ortega et al. 1993; Barriga et al. 2002). This effect on the innate response is much clearer than those observed in previous studies without RosA (Ortega et al. 2017; Ortega-Collazos et al. 2024), supporting the use of this polyphenolic acid in the context of mud-bath interventions. Thus, in our view, the observed stimulation of phagocytic and microbicidal activity is most likely an indirect effect mediated by RosA, attributable to its anti-inflammatory, antioxidant, and immunoneuroendocrine-stabilizing properties. These systemic effects may improve the functional environment of neutrophils, thereby enhancing their capacity, rather than being the result of a direct action, as no previous in vitro studies have demonstrated that RosA directly stimulates these functions in neutrophils. Additionally, the hormetic increase in systemic cortisol concentration observed in this context may also directly contribute to the stimulation of the neutrophil-mediated innate immune response. In any case, the significant enhancement of innate immunity observed with the application of a RosA-enriched peloid opens new avenues for investigating the use of this polyphenolic acid in mud-bath therapies for the elderly, who are often immunosuppressed and more vulnerable to infections.

In the context of osteoarthritis and pelotherapy, RosA-fortified peloids did not exhibit reduced efficacy in improving mobility and pain compared to non-fortified formulations. On the contrary, they appeared to confer additional mental health benefits, including reduced anxiety and an increased proportion of cells displaying innate immune responsiveness (Ortega-Collazos et al. 2024). These effects are closely linked to the stabilization of the immunoneuroendocrine response and to the bioregulatory, hormetic properties of balneotherapy, primarily mediated by IL-8 and cortisol (Gálvez et al. 2018a, b). While the decrease in IL-8 may be partly due to cortisol increases triggered by hyperthermia-induced hormetic stress, the inclusion of RosA could further enhance the anti-inflammatory potential of the intervention. This may occur through RosA’s capacity to suppress IL-8 production (Luo et al. 2020) and alleviate anxiety by modulating the hypothalamic-pituitary-adrenal axis and upregulating glucocorticoid receptor expression in the hippocampus (Makhathini et al. 2018). Furthermore, elevated glucocorticoid levels may contribute to stimulating neutrophil-mediated innate immune activity (Ortega 2016; Gálvez et al. 2018a).

The findings related to IL-6 are more nuanced. Although its role in osteoarthritis remains debated, elevated IL-6 levels have been consistently associated with cartilage degradation, disease severity, and pain (Livshits et al. 2009; Stannus et al. 2010; Galvez et al. 2017). In our study, however, baseline IL-6 concentrations were approximately 3 pg/mL—considerably lower than those reported in earlier studies—suggesting an absence of significant systemic inflammation. While the post-intervention increase in IL-6 may reflect a hormetic response contributing to immunoneuroendocrine regulation, it likely lacks clinical relevance, as levels remained below pro-inflammatory thresholds. Notably, in patients with higher inflammatory profiles, similar peloid-based interventions have demonstrated significant reductions in IL-6 (Ortega et al. 2017).

## Conclusions

In conclusion, the hyperthermic mud-bath intervention with a peloid enriched with RosA induces immuno-neuroendocrine stabilization, mediated by an increased physiological release of cortisol and a decrease in IL-8 concentration, alongside a strong stimulation of the innate phagocytic and microbicidal response of neutrophils. These neuroimmune and bioregulatory responses underlie the clinical benefits—reduced pain and increased mobility—induced by the intervention, which are also reflected in a reduction in the use of analgesics by patients. These findings also open new avenues for utilizing RosA in the context of mud therapy for rheumatic and inflammatory diseases.

## Limitations of study

Although the study population may appear limited, this research is not intended to be a clinical trial—despite presenting clinical data that confirm the well-documented benefits of El Raposo, S.L. peloids on functional capacity and pain. Rather, the study aims to further explore the mechanisms underlying their effectiveness, particularly within the immunoneuroendocrine response framework. In this context, the sample size and statistical power are deemed sufficient based on previous experience.

Another potential limitation is the absence of a control group using a peloid without RosA. Nonetheless, this was not considered essential given the extensive literature already available on standard peloids. Prior studies have shown that the therapeutic benefits of the RosA-enhanced peloid are not inferior to those of the unenhanced version. These findings now pave the way for future investigations into whether the enhanced effects on the innate immune response are due to direct actions of RosA or to indirect mechanisms mediated by immunoneuroendocrine stabilization. Naturally, longer intervention periods or extended post-intervention follow-up assessments may also provide additional, and particularly clinically relevant, insights in future studies.

## Data Availability

The participants of this study did not give written consent for their data to be shared publicly, so data is unavailable due to privacy or ethical restrictions. Data supporting the reported results are stored confidentially in the Research Group Archives.
